# A Case of Autoimmune Polyglandular Syndrome Type 2 in Childhood: Unraveling a Rare and Complex Endocrine Puzzle

**DOI:** 10.7759/cureus.87595

**Published:** 2025-07-09

**Authors:** Felicita M Tayong, Faiza Gul, Kasagani Srujana, Phillip George, Aysha Habib, Nadia Khan, Nida Gul, Ayaz Ali

**Affiliations:** 1 General Surgery, Tulane University School of Medicine, New Orleans, USA; 2 College of Medicine, University of Science, Arts and Technology, Olveston, MSR; 3 Paediatrics, Lady Reading Hospital Peshawar, Peshawar, PAK; 4 Pediatrics, Government Medical College, Siddipet, Siddipet, IND; 5 Internal Medicine, Ross University School of Medicine, Bridgetown, BRB; 6 Internal Medicine, Saidu Medical College, Saidu Sharif, PAK; 7 Internal Medicine, Khyber Teaching Hospital Peshawar, Peshawar, PAK; 8 Medicine, Lady Reading Hospital Peshawar, Peshawar, PAK; 9 Internal Medicine, Khyber Medical College Peshawar, Peshawar, PAK

**Keywords:** aps type ii, endocrinology, hypothyroidism, paediatric endocrinology, severe hypoglycemia, type i diabetes mellitus

## Abstract

Autoimmune polyendocrine syndrome type 2 (APS-2) is a rare autoimmune disorder characterized by the coexistence of multiple endocrine gland dysfunctions, most commonly type 1 diabetes mellitus (T1DM), autoimmune thyroid disease, and Addison’s disease. It typically manifests in middle-aged women, with pediatric cases being exceedingly uncommon. We present the case of a nine-year-old boy with a known history of T1DM diagnosed at the age of six years, who presented to the emergency department with severe hypoglycemia and was subsequently found to have severe hypothyroidism. Clinical features included growth retardation, coarse facial features, alopecia, developmental delay, and hepatomegaly. Laboratory investigations confirmed severe hypothyroidism alongside his known T1DM, fulfilling the diagnostic criteria for APS-2. The patient was managed with levothyroxine replacement and close glycemic monitoring. This case highlights the importance of considering APS-2 in pediatric patients with multiple autoimmune conditions, emphasizing the need for early recognition and comprehensive endocrine evaluation to guide appropriate management and improve long-term outcomes.

## Introduction

Multiple endocrine gland dysfunction is a rare set of immune-mediated illnesses known as autoimmune polyendocrine syndrome (APS) [1]. The most recent illness taxonomy divides APS into three primary subtypes: IPEX (immune dysregulation, polyendocrinopathy, enteropathy, X-linked), APS-1, and APS-2 [2]. APS-2 is defined by the coexistence of two or more endocrine disorders, commonly including type 1 diabetes mellitus (T1DM), autoimmune thyroid disorders (AITD), Addison’s disease, or other autoimmune conditions. It usually appears in young adulthood and starts later than APS-1 [3,4].

Genetic vulnerability to APS-2 is largely determined by the human leukocyte antigen (HLA) gene complex, with HLA-B8 and DR3 alleles being highly linked to a higher chance of APS-2 development [5,6].

APS type 2 occurs infrequently in children and is most frequently observed in middle-aged women. Each syndrome component's diagnosis is verified by looking for disease-specific immunological antibodies. Antibody testing may help predict the future emergence of autoimmune endocrine disorders because families with polyglandular autoimmune syndrome Type II (PAS II) often have silent autoantibodies [7].

This report describes the case of a nine-year-old boy who was diagnosed with APS2, T1DM first, and hypothyroidism later. By documenting this case, we hope to contribute more clinical information to advance early diagnosis and treatments and enhance knowledge of the uncommon features of APS-2 in children.

## Case presentation

A nine-year-old male child was brought to the Pediatric Emergency Department in an unconscious state. The child was a known case of T1DM, diagnosed at the age of six years, and was being managed with a basal-bolus insulin regimen prescribed by an endocrinologist.

Upon arrival, random blood sugar (RBS) was checked and found to be 32 mg/dL, indicating severe hypoglycemia. Immediate resuscitative measures were undertaken. Intravenous (IV) access was secured, and the child was administered 10% dextrose at a dose of 5 mL/kg. Given the child’s weight of 14 kg, a total of 70 mL of 10% dextrose was infused as an IV bolus.

Further history obtained from the mother revealed that the child had developmental delays. On physical examination, the child appeared pale with coarse facial features, alopecia, and edema. Anthropometric measurements were as follows: weight 14 kg, height 92 cm, occipitofrontal circumference (OFC) 43 cm. All parameters were below the third percentile for age, with the OFC indicating microcephaly. Hepatomegaly was also noted on abdominal examination. The details of investigations done are given in Table 1.

**Table 1:** Laboratory test results T3: triiodothyronine; T4: thyroxine

Tests	Patient Value	Normal Range	Interpretation
Random Blood Sugar	32 mg/dl	70-140 mg/dl	Severe hypoglycemia
Complete Blood Count			
Total leukocyte count	18,000/mm³	4,000 – 11,000/mm³	Leukocytosis
Hemoglobin	9 g/dL	11.5 – 15.5 g/dL (for 9-year-old)	Anemia
Renal Function Test			
Serum creatinin	0.4 mg/dL	0.3 – 0.7 mg/dL	Normal
Blood urea nitrogen	9 mg/dL	5 – 18 mg/dL	Normal
Liver Function Test			
Aspartate transaminase	30 U/L	AST < 50 U/L	Normal
Alanine transaminase	26 U/L	ALT < 40 U/L	Normal
Bilirubin	0.4 mg/dL	0.2 – 1.2 mg/dL	Normal
Serum calcium	8.7 mg/dL	8.5 – 10.5 mg/dL	Normal
Serum phosphorus	4.6 mg/dL	4.5 – 6.5 mg/dL	Normal
Thyroid Function Test			
Thyroid-stimulating hormone	>150 µIU/mL	0.5 – 4.5 µIU/mL	Markedly elevated
T3	<10 ng/dL	80 – 200 ng/dL	Severely low
T4	<0.3 µg/dL	5 – 12 µg/dL	Severely low

Celiac disease screening was negative. Cortisol levels were within normal limits, and the adrenocorticotropic hormone (ACTH) stimulation test was also unremarkable, effectively ruling out adrenal insufficiency.

**Figure 1:**
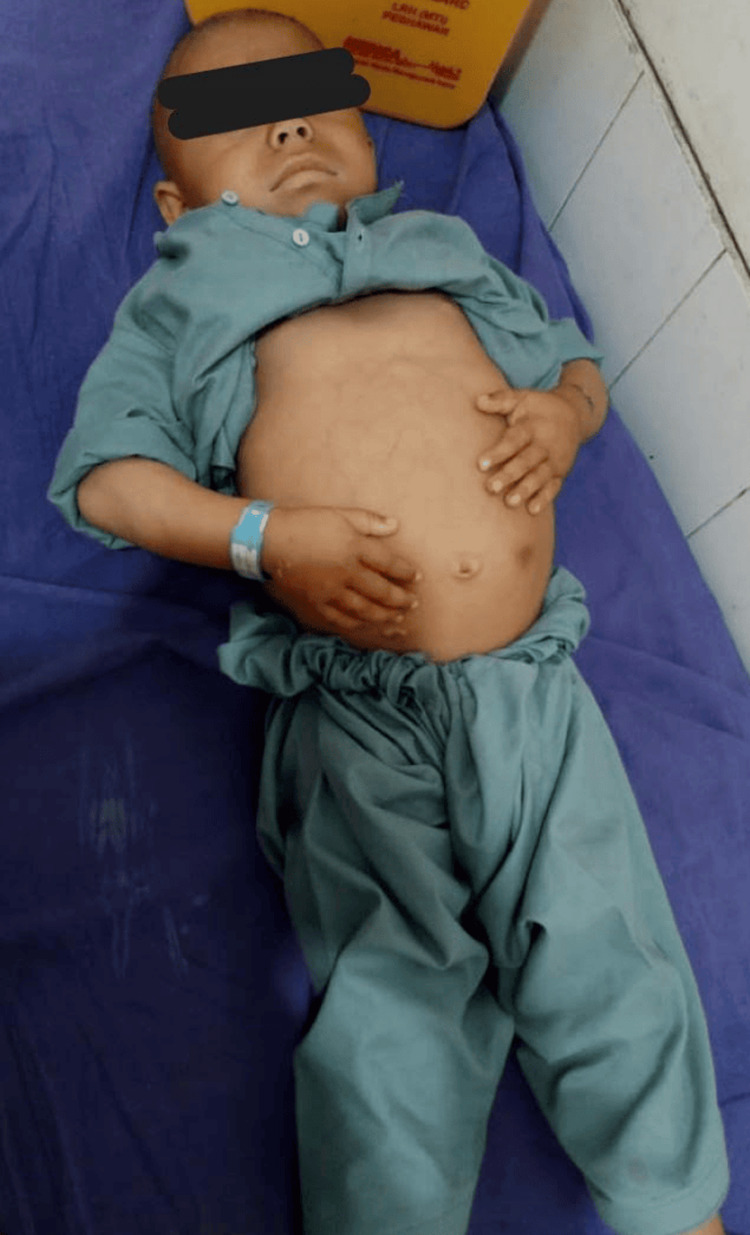
Image of the patient showing a distended abdomen Written and signed consent for use of photograph has been provided by parents

An abdominal ultrasound was performed to further evaluate the hepatomegaly observed on clinical examination. Ultrasound imaging showed a distended abdomen (Figure 1) with increased gas content and an enlarged left hepatic lobe. The diameter of the portal vein remained within normal parameters. Both the gallbladder and spleen appeared normal in size and echotexture, with no focal lesions or abnormalities detected.

**Figure 2:**
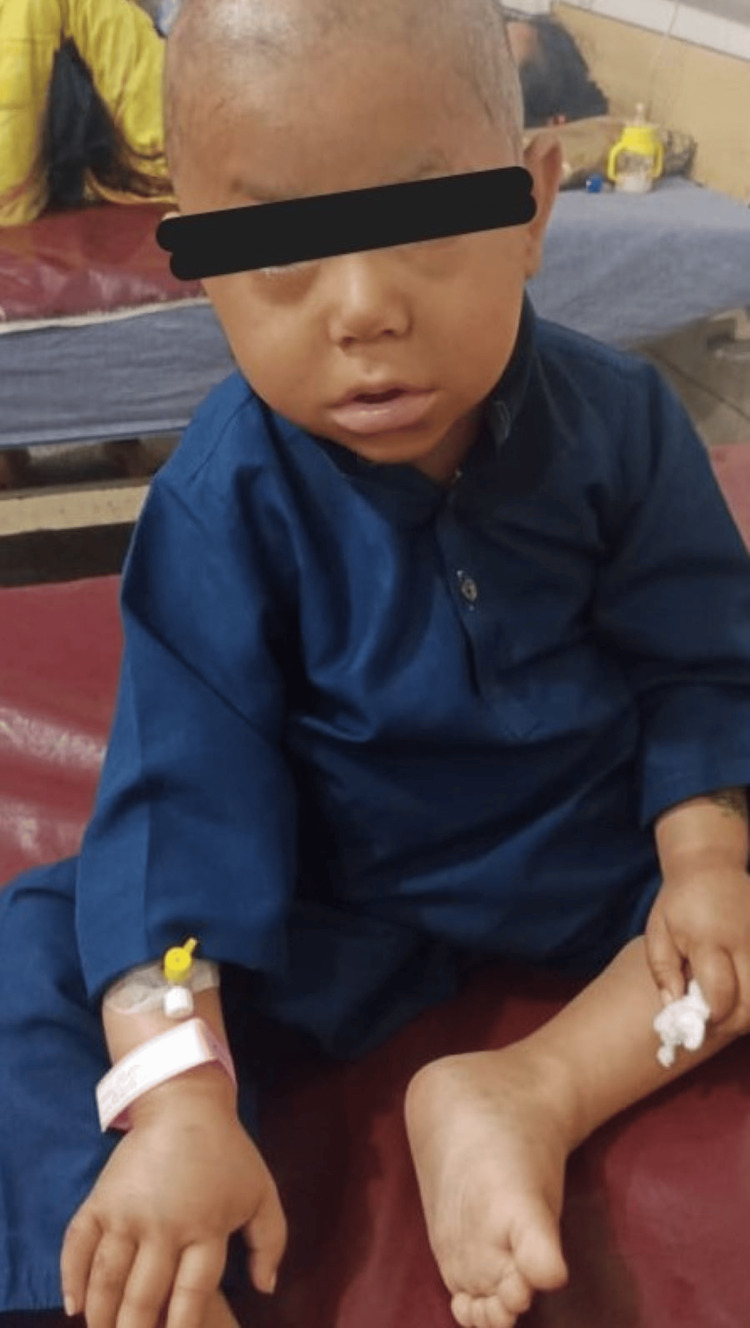
Image of the patient showing coarse facies Written and signed consent for use of photograph has been provided by parents

In view of the child’s long-standing T1DM, recent episode of severe hypoglycemia, growth retardation, coarse facies (Figure 2), developmental delay, and newly diagnosed severe hypothyroidism, a multisystem autoimmune process was considered. The combination of these endocrine abnormalities was consistent with a diagnosis of APS-2. This diagnosis was based on the co-occurrence of T1DM, autoimmune hypothyroidism, and clinical features suggestive of chronic endocrine dysfunction.

The patient was commenced on thyroid hormone replacement therapy with levothyroxine (thyroxine) to manage hypothyroidism and restore euthyroid status. In addition, regular monitoring of random blood sugar levels was initiated to assess and manage any potential glycemic disturbances.

## Discussion

APS-2, also referred to as Schmidt’s syndrome, is characterized by the coexistence of two or more autoimmune conditions. These typically include primary adrenal insufficiency (Addison’s disease), autoimmune thyroid disorders such as Graves’ disease or primary hypothyroidism, T1DM, celiac disease, and pernicious anemia. The mineralocorticoid fludrocortisone is often used in the management of adrenal insufficiency associated with this syndrome [8-10].

APS-2 affects approximately one in 20,000 individuals, with a higher prevalence among females, showing a male-to-female ratio of 1:3. The syndrome most commonly presents between the ages of 20 and 60 years [11]. The development of APS-2 involves a combination of genetic predisposition and environmental influences, with a prolonged subclinical phase of tissue destruction often preceding the clinical manifestation of the syndrome. The inheritance pattern is multifactorial, with a significant contribution from genes located on chromosome 6 and a notable association with certain HLA alleles, particularly DR3 and B8. The fact that monozygotic twin concordance is below 100% suggests the involvement of additional non-genetic factors. Furthermore, environmental and epigenetic triggers, such as viral or bacterial infections, hormonal factors, nicotine use, and psychosocial stressors, may initiate or exacerbate the autoimmune cascade linked to DR3. However, exposure to these factors alone does not uniformly result in disease development [12,13].

Patients with APS-2 may present with a wide range of clinical symptoms, such as loss of appetite, persistent fatigue, nausea, vomiting, generalized muscle weakness, abdominal pain, and episodes of diarrhea. In cases where adrenal insufficiency is present, features may include hyperpigmentation of the skin and mucous membranes, low blood sugar levels, orthostatic hypotension, and, in severe situations, adrenal crisis characterized by shock-like symptoms. When T1DM is part of the syndrome, patients often experience excessive urination (polyuria), increased thirst (polydipsia), and elevated blood glucose levels. In individuals with hypothyroidism, signs can include a slowed heart rate (bradycardia) and delayed relaxation of deep tendon reflexes [14].

The primary approach to managing APS-2 focuses on hormone replacement to correct the specific deficiencies, similar to the treatment of isolated endocrine disorders. It is crucial to initiate adrenal corticosteroid therapy prior to starting thyroid hormone replacement in order to avoid precipitating an Addisonian crisis [15].

Three years after his initial diagnosis of T1DM, the patient was found to have hypothyroidism when he presented to the emergency department with hypoglycemia related to T1DM. The presence of these two autoimmune conditions led to the diagnosis of APS-2.

## Conclusions

Although APS-2 is rare in children, it should be considered in young patients presenting with multiple endocrine abnormalities. Early recognition is essential to prevent life-threatening complications such as severe hypoglycemia or adrenal crisis. This case underscores the need for clinicians to maintain a high index of suspicion for APS-2 in pediatric patients with T1DM and newly emerging autoimmune features. Prompt diagnosis and appropriate hormonal replacement therapy can significantly improve clinical outcomes and quality of life in these patients.
